# Addressing the Global Influence of Unethical Formula Marketing

**DOI:** 10.9745/GHSP-D-22-00120

**Published:** 2022-04-28

**Authors:** Cecília Tomori

**Affiliations:** a Johns Hopkins School of Nursing, Baltimore, MD, USA.; b Department of Population, Family and Reproductive Health, Johns Hopkins Bloomberg School of Public Health, Baltimore, MD, USA.

## Abstract

Projects that seek to address Code implementation need to understand the current landscape of corporate marketing activities in the government and health care settings and mobilize stakeholders from the government to the community when implementing these projects.

See related article by Reinsma et al.

Unethical marketing of commercial milk formulas remains a global challenge.^1^ Despite the establishment of the Code more than 40 years ago, steps for its implementation remain inadequate. Few nations have adopted the Code in their legal frameworks, and the monitoring and enforcement of the laws and regulations remains inadequate. As a result, rampant violations of the Code continue, undermining breastfeeding and putting hundreds of lives at risk each year.^2^^,^^3^ The recent large World Health Organization (WHO) and UNICEF study^1^ undertaken in 8 countries with thousands of participants demonstrates that formula companies continue to have a pervasive influence on infant feeding decisions. They deploy sophisticated, emotionally resonant messages through multiple channels to undermine confidence in breastfeeding and to capture consumers. Distorting science is a key part of their strategy to convince parents as well as health professionals. The report's findings demonstrate the importance of efforts to enforce the Code so that families can make informed decisions.

In a recent issue of *GHSP*, Reinsma et al.^4^ describe the evaluation of a project in the Philippines that aimed to address the gap in monitoring by building a platform that enabled reporting Code violations. As part of the project, the team provided outreach to a wide range of people including members of the community, community health workers, health care providers, breastfeeding advocates, and government agencies about the laws to help prepare for reporting violations using newly developed platforms, including a website, an app, and free short message service (SMS) messages. The project then evaluated the reporting system. The team found several improvements in feeling more informed about the laws and empowered by the reporting system. At the same time, their findings also indicated several challenges, including the preference for in-person resolution of violations over a digital platform and concerns over confidentiality. Moreover, coordinating with government stakeholders proved complex. The team found the coordination challenging and time consuming, and even after launching the project, few reports of violations were addressed.

While some of the challenges are due to limited resources, many complexities Reinsma et al.^4^ describe are embedded in deeper histories. To better understand these challenges, we must examine the long history of formula companies' efforts to assert influence. Nestlé's marketing efforts in the Philippines date back to 1895 and the company continues to play a dominant role, sharing the vast majority of the market with Reckitt Bensicker, which produces Mead Johnson.^5^ Aggressive marketing has continued even after the adoption of the WHO Code into laws. Indeed, systematic efforts to undermine breastfeeding by companies remain the norm, rather than the exception. For instance, in 2017 Mead Johnson targeted health professionals directly with claims about one of its products to circumvent advertising regulations. This was a highly successful strategy, leading to a substantial increase in sales (40%) over 3 months.^6^^,^^7^ Such aggressive marketing efforts are coupled with extensive corporate activities undertaken to influence laws and implement marketing practices that subvert regulations and undermine breastfeeding.

Systematic efforts to undermine breastfeeding by companies remain the norm, rather than the exception.

As described in detail by Baker et al.,^5^ formula companies in the Philippines have engaged in extensive efforts to undermine marketing regulations, relying on multipronged efforts. A key component was establishing a corporate front group with other formula manufacturers called the Infant and Pediatric Nutrition Association of the Philippines (IPNAP), which participates widely in important meetings about infant and child nutrition and has carried our extensive lobbying activities. For instance, IPNAP lobbied to pass legislation in 2012 that was framed as helpful for nutrition, but, in reality, it undermined existing breastfeeding protections by facilitating formula donations during disasters without prior approval, allowing marketing in health facilities, and enabling companies to carry out health professional education. IPNAP had an extremely close relationship with the government with a former Department of Health employee and Congressman at its helm. While the 2012 legislative effort was unsuccessful, in the past 10 years, corporate lobbying activity continued, including efforts to distribute formula during disasters. This is particularly galling since the Philippines experiences a frequent number of disasters from weather and volcanic activity in the region and the harms of formula distribution in these situations are well documented.^8^^,^^9^

The activities mentioned here are just a selection of corporate political activities undertaken in the Philippines. In light of this history, it is not surprising that coordinating with the government proved challenging. Political will is necessary to succeed in fully implementing and enforcing laws. Unfortunately, corporate actors play an enormous role in undermining them. Of course, such practices are hardly unique across settings.^10^

Reinsma et al.^4^ demonstrate the challenges of enforcing the Code even when legal frameworks are supportive. Without adequate systems that work with and for local communities, laws remain insufficient. To address these issues, projects aiming to enhance Code implementation need to closely examine the history of corporate activities in government organizations and health care settings and develop a sophisticated understanding of the current landscape of these efforts. Mobilizing stakeholders within the government as well as within public health and community organizations needs to be the foundation of implementation efforts. Any reporting system must have consequences—if reports remain unaddressed, they become futile. Throughout this process, as Reinsma et al. point out, it's essential to listen to local communities' preferences for how they wish to be engaged in Code implementation efforts. Any effort must prioritize confidentiality to avoid reprisals, and technological solutions may not always be best suited to achieve aims. What may work in one context may not be appropriate in another. Together, these findings also suggest that prioritizing local knowledge and expertise can not only help achieve immediate aims but also make them more sustainable in the long run while also taking up calls from the Global South to decolonize and address the power imbalances in the infrastructure of global health.^11^

It's essential to listen to local communities' preferences for how they wish to be engaged in Code implementation efforts.
